# Post-neoadjuvant chemoradiotherapy tumor resectability following induction chemotherapy in locally advanced proximal gastric and adenocarcinoma of the esophagogastric junction: A clinical trial

**DOI:** 10.22088/cjim.12.3.256

**Published:** 2021-04

**Authors:** Seyed Amir Aledavood, Kazem Anvari, Soodabeh Shahidsales, Sare Hosseini, Ali Emadi Torghabeh, Masume Masudian

**Affiliations:** 1 Cancer Research Center, Mashhad University of Medical Sciences, Mashhad, Iran; 2 Kosar Hospital, Semnan University of Medical Sciences, Semnan, Iran

**Keywords:** Neoadjuvant chemoradiotherapy, Proximal gastric adenocarcinoma, Esophagogastric junction (EGJ) adenocarcinoma

## Abstract

**Background:**

Pre-operative chemoradiotherapy (NACRT) of patients with proximal gastric and esophagogastric junction (EGJ) adenocarcinoma may result in increased local control and improved patients’ survival rate. This study aimed to investigate the effect of NACRT on resectability of tumor in patients with proximal gastric and EGJ adenocarcinoma.

**Methods:**

In this single-arm clinical trial, patients with locally advanced proximal gastric and EGJ adenocarcinoma were included. Two courses of paclitaxel/carboplatin chemotherapy alone followed by NACRT with a similar treatment regimen and a total radiation dose of 45-50.4/1.8-2 Grays were prescribed. After surgery, patients were evaluated for resection rate, pathologic response rate, and post-surgical complications.

**Results:**

A total of 61 patients with a mean age of 65.9 years participated. Grades 1 and 2 were the most prevalent side effects, with grade 3 being the worst grade and exhibiting as leukopenia (4.9%) and thrombocytopenia (1.6%). 25 (41%) patients underwent surgery after NACRT. Post-surgery complication was reported in 20% of cases (including 8% mortality and 12% morbidity). R0 and R2 resection was observed in 88% and 12% of cases, respectively. Complete pathologic-response was achieved in 24% of patients.

**Conclusion:**

Paclitaxel/carboplatin based neoadjuvant chemotherapy was associated with potential resectability and appropriate pathologic response in patients with locally advanced proximal gastric and EGJ adenocarcinoma. However, by reducing patient tolerance to complete courses of weekly chemotherapy, induction chemotherapy lowered the effectiveness of concurrent chemotherapy and radiotherapy (as a sensitizing agent). Hence, induction chemotherapy proved to be more unbeneficial causing delayed treatment and reducing concurrent chemoradiotherapy tolerance.

Gastric cancer is one of the leading causes of cancer-related deaths worldwide. Its incidence and prevalence are various in different parts of world, however, the highest rates are reported in East Asia, East Europe, and South America and lowest in North America and Western Europe (1). In Iran, it is the second common cancer and associated with high mortality rate (2, 3).

Various protocols are recommended by different cancer organization for the treatment of gastric cancer. Nevertheless, in most practices, initial surgery is recognized as standard of treatment (4). Surgery in early stages proves curative, however gastric cancers are often diagnosed in advanced stages. Therefore, the adjuvant treatments are recommended. In the meantime, large number of patients (approx. 50%) are discovered inoperable during surgery and technically advanced surgical procedures do not appear capable of significantly altering the clinical results. On the contrary, post-operative adjuvant chemoradiotherapy, has proven beneficial in reducing the risk of local recurrence. However, its pre-operative efficacy remains to be explored and decisively confirmed as a standard procedure (4-7).

The main advantages of neoadjuvant chemotherapy include tumor size reduction for easier surgical removal, improved sterility of surgical site, and reduced risk of cancer cells shedding into peritoneal cavity amid stomach handling in the operating room (7). Accompanied by possible reduction in the volume of radiation-exposed intestine, neoadjuvant chemoradiotherapy is better tolerated than many other post-surgical treatments. Additionally, tumor response to neoadjuvant chemoradiotherapy is enhanced as a result of preoperative blood and oxygen delivery (8). 

In majority of cases, assessment of efficiency and applicability of neoadjuvant chemoradiotherapy is based on phase-2 trial evaluations. In their study, *Wydmański et al*. (2007) evaluated the effects of preoperative chemoradiotherapy on 40 patients with operable gastric cancer using 45 gray (Gy) radiation to the stomach and regional lymph nodes and concurrent 5-fluorouracil (5-FU) and leucovorin. Study results revealed an improved pathologic response rate and R0 resection together with reduced local recurrence and increased 2-year survival rate (9). Alternately, studies conducted by *Burmeister et al.* (10), *Walsh et al*. (11), *Van Hagen et al*. (12), *Urba et al*. (13) support the superiority of trimodality treatment results and preference of administering neoadjuvant treatment as a favored approach. In a phase 2 study, *Aledavood et al*. assessed the neoadjuvant chemoradiotherapy using 5-FU and leucovorin. The results were promising, although a change in the proposed treatment protocol and regimen appears effectual in terms of end results (14). The current clinical trial is designed to evaluate the effects of induction chemotherapy and preoperative chemoradiotherapy on post-operative tumor response rate and its resectability. 

## Methods

This is an open labelled single-armed phase-2 clinical trial conducted on 61 patients with proximal gastric and esophagogastric junction (EGJ) adenocarcinoma referring to the 2016-2018 period in Radiation Oncology Department of Omid and Emam Reza Teaching Hospitals affiliated to Mashhad University of Medical Sciences.

Inclusion criteria were the pathologically and endoscopically confirmed proximal gastric and EGJ adenocarcinoma, Siewert type II and III tumors on initial endoscopy/EUS, in addition to age requirement of >18 and Karnofsky performance status index>60%. AJCC staging system was used to define clinical T3-T4 and/or node positive tumors. Moreover, normal baseline cell blood count, liver and kidney tests, and fasting blood sugar test results were required to be included. Exclusion criteria encompassed prior history of chemotherapy, prior history of significant comorbidities (e.g. uncontrolled hypertension, uncontrolled diabetes, liver failure, renal failure, cardiovascular diseases, and conditions causing uncontrolled bleeding), concurrent presence of other cancers (apart from proximal gastric/EGJ), prior history of hypersensitivity to paclitaxel medication, pregnancy, and patient reluctance to alopecia and surgery. 

The protocol of study was approved by the Mashhad University of Medical Sciences Ethics Committee with ref. no. IR.MUMS.FM.REC.1396.03 and registered at Iranian Registry of Clinical Trials with IRCTID: IRCT2017070834945N1. 

After obtaining written informed consent form, patients were staged by endoscopic ultrasound (EUS) and thorax, abdominal, and pelvic CT scan. Treatment began one to two courses of paclitaxel/carboplatin chemotherapy sessions followed by concurrent chemoradiotherapy with 175mg/m^2 ^of paclitaxel and AUC 5 carboplatin doses every 3 weeks. Concurrent chemotherapy and radiotherapy followed the same with weekly protocol. Radiotherapy was given a total dose of 45-50.4/1.8-2 with LINAC 6-15 MV photons to gastric and paraesophageol lymphatic, paragastric, celiac, splenic, and suprapancreatic regions with the volume of CTV recieving an acceptable 95-100% of the target dose on a single daily session/5 days a week. 4-6 weeks after treatment completion, patients were re-examined for distant metastasis. Patients without reported metastasis underwent surgery. Surgical and pathological findings were recorded to help determine the extent of resection , as well as tumor response rate to neoadjuvant treatment. Patients were monitored for side effects. Principal variables studied included pathologic response based on results of pathologic examination of surgical specimen ("complete pathologic response denoting absence of tumor cell in the original tumor site and lymphatic nodes; "relatively responsive" denoting limited presence of tumor accompanied with fibrosis in the original site and examined nodes; "unresponsive" denoting presence of tumor cells in the original tumor site and/or lymphatic nodes), surgical margin according to (R)-staging based on findings during surgery and pathologic report ("R0" denoting absence of residual tumor at the resection margin; "R1" denoting presence of microscopic residual tumor; "R2" denoting presence of gross residual tumor), tumor stage as evaluated by AJCC cancer staging system (7th edition), and treatment side effects measured using Common Terminology Criteria for Adverse Events (CTCAE) scale (EORTC grade 1-5). Descriptive analysis was done by means of SPSS®-v21 statistical analysis software. 

## Results

**Fig 1:**
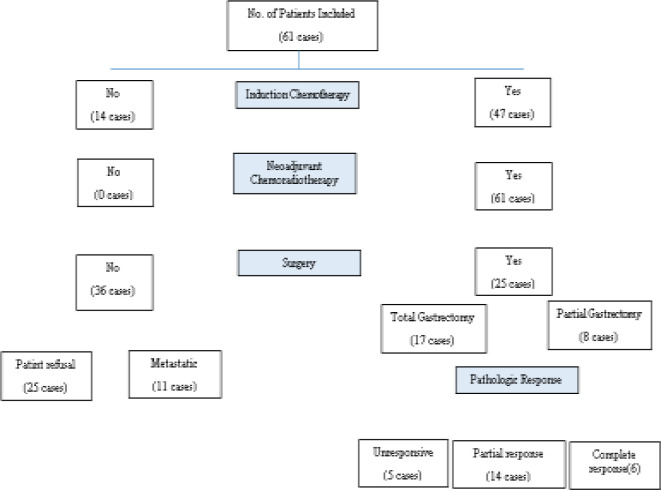
Flowchart of patients studied

A total of 61 patients with a mean age of 65.9 years and a satisfactory performance status (85% with KPS>70) were included in the study (Figure 1).

Of the overall 61 patients studied, 22 (33.1%) underwent EUS indicating a cT3 for 72.7% and cT4 for 22.7% of patients. Lymph node involvement was positive in 27.9% and 95.4% of patients based on CT and EUS results, respectively (table 1). 

**Table 1:** Background specifications of test subjects at the beginning stage

Specification	Study Group **N=61**
**Demographic**	
**65.93** **±** **10.13 (66) years**	Age (**mean**)
	Gender
**50 (83.3%)**	**male**
**11 (16.7%)**	**female**
**22.69±3.78 (22.6)**	Body Mass Index (**mean**)
	Performance Status (KPS)
**9 (14.7%)**	**60**
**29 (47.5%)**	**70**
**2 (3.3%)**	**80**
**21 (34.4%)**	**90**
**Tumor Related**	
	Degree of Differentiation
**9 (14.7%)**	**Well differentiated**
**23 (37.8%)**	**Intermediate differentiated**
**23 (37.8%)**	**Not differentiated**
**6 (9.8%)**	**Not Reported**
	Clinical EUS in T (22 cases)
**1 (4.5%)**	**CT2**
**16 (72.7%)**	**CT3**
**5 (22.7%)**	**CT4**
	Clinical EUS in N (22 cases)
**1 (4.5%)**	**CN0**
**7 (31.8%)**	**CN1**
**11 (50%)**	**CN2**
**3 (13.6%)**	**CN3**
	N based on CT Scan (48 cases)
**17 (27.9%)**	**N+**
**Work Up Related**	
	**Systemic**
**13 (21.3%)**	**Sonography**
**48 (78.7%)**	**CT Scan**
	**Local**
**61 (100%)**	**Endoscopy**
**22 (36.1%)**	**EUS**

Induction chemotherapy was delivered to 47 (77%) patients prior to radiotherapy. The entire patient population received concurrent chemoradiothrapy due to low tolerance and performance of patients ,44.3% of them received only one to three weekly courses of chemothrapy concomitant with radiothrapy. Chemoradiotherapy was administered with a dose of 50.4 gray in frequent cases (29 or 47.5%). Upon completion of chemoradiotherapy, 25 (41%) patients underwent surgery. No surgery was applied to the remaining population owing to patient reluctance (25 or 69.6%) and appearance of metastasis before surgery (11 or 30.5%). Data related to each treatment type is presented in the table (table 2). 

**Table 2:** Details of treatments applied to test subjects

Specification	Study Group **N=61**
Induction Chemotherapy	
	Prescription
**47 (77%)**	**Yes**
**14 (23%)**	**No**
	No. of Courses (weekly)
**25 (53.1%)**	**1-3**
**13 (27.7%)**	**4 and 5**
**9 (19.1%)**	**6 to 10**
Concurrent Chemotherapy	
	Prescription
**61 (100%)**	**Yes**
**0 (0%)**	**No**
	No. of Courses (weekly)
**7 (11.5%)**	**1**
**7 (11.5%)**	**2**
**13 (21.3%)**	**3**
**16 (26.2%)**	**4**
**12 (19.7%)**	**5**
**6 (9.8%)**	**6**
Radiotherapy	
	Prescription
**61 (100%)**	**Yes**
**0 (0%)**	**No**
	Dose
**18 (29.5%)**	**46 gray**
**36 (59%)**	**46-50 gray**
**7 (11.5%)**	**More than 50 gray (50.4-54)**

**36 (59%)**	**No**
**25 (41%)**	**Yes**
**17 (68%)**	**Total Gastrectomy**
**8 (32%)**	**Partial Gastrectomy**

For reason of data conformity, chemotherapy courses are presented on weekly basis with each single paclitaxel + carboplatin tri-weekly course being equivalent to 3 paclitaxel + carboplatin courses per week. Grade 3-4 leukopenia (4.9%) and thrombocytopenia (1.6%) were the most common side effects. Other side effects were grades 1 and 2. Side effects related to the adjuvant chemotherapy treatments applied in this study are presented in the table (table 3). 

**Table 3:** Adjuvant chemotherapy side effects in this study

Treatment Toxicity	Study Group **N=61**
Hematological toxicity	
	Leukopaneia
**26 (42.6%)**	**Grade 1**
**32 (52.5%)**	**Grade 2**
**3 (4.9%)**	**Grade 3**
**0 (0%)**	**Grade 4**
	Neutropenia
**48 (78.8%)**	**Grade 1**
**13 (21.3%)**	**Grade 2**
**0 (0%)**	**Grade 3**
**0 (0%)**	**Grade 4**
	Anemia
**50 (82%)**	**Grade 1**
**11 (18%)**	**Grade 2**
**0 (0%)**	**Grade 3**
**0 (0%)**	**Grade 4**
	Thrombocytopenia
**59 (96.7%)**	**Grade 1**
**1 ()1.6%-**	**Grade 2**
**1 (1.6%)**	**Grade3**
**0 (0%)**	Grade 4
Non hematologic Toxicity	
	Nausea
**54 (88.5%)**	**Grade 1**
**7 (11.5%)**	**Grade 2**
**0 (0%)**	**Grade 3**
**0 () 0%**	**Grade 4**
	Vomit
**51 (83.6%)**	**Grade 1**
**10 (16.4%)**	**Grade 2**
**0 (0%)**	**Grade 3**
**0 (0%)**	**Grade 4**
	Anorexia
**30 (50%)**	**Grade 1**
**30 (50%)**	**Grade 2**
**0 (0%)**	**Grade 3**
**0 (0%)**	**Grade 4**

The main outcomes considered by this study are the extent of resection and tumor response to neoadjuvant treatment based on the result of pathologic examination. Out Of the 25 (41%) cases receiving surgery, 88% (22 cases) exhibited R0 and the remaining 12% (3 cases) exhibited R2 resection. Major post-surgery events (including 1-month mortality and complications requiring repeated surgery) were reported in 20% of operated patients and involved 8% mortality (2 deaths two weeks after surgery) and 12% morbidity (3 cases of anastomotic leak and fistula). All anastomotic leak and fistula cases recovered after surgical repair based on pathologic evaluation for neoadjuvant effect, 24% (6) cases of patients have complete pathologic response. 5 patients were unresponsive and 14 were relatively responsive. 

## Discussion

This study was carried out with the aim of investigating the role of preoperative oncologic treatment role of preoperative oncologic treatment and evaluating the pathalogic response rate of patients with proximal gastric and EGJ adenocarcinomas to neoadjuvant treatment. Protocols and results of studies related to neoadjuvant treatment of patients with locally advanced proximal gastric and EGJ adenocarcinomas is summarized in the table (table 4). 

**Table 4:** Studies on neoadjuvant treatment of patients with locally advanced proximal gastric and esophagogastric junction (EGJ) adenocarcinomas

PCR* **%**		R0* **%**	**n (** **total** **)** **(SWI&II)**	**Radioptherapy**	**Chemotherapy during CRT**	**Pre-CRT Chemotherapy**	**Method**	**Study**
**20**	**82**		**41**	**45-50.4 Gy/ 1.8-2 G**	**cape or 5FU**	**-**	**CT**	**Aledavood (14)**
**26**	**77**		**49**	**45 / 1.8 G**	**5FU+pacli**	**5FU+leu+cis**	**CT**	**...** ** (19)**
**11**	**95**		**24**	**45 / 1.8 G**	**5FU**	**-**	**Pilot**	**… (20)**
**19**	**86**		**48**	**21.6-50.4 (med:45 Gy**	**Varied**	**-**	**Retro**	**… (18)**
**17.5**	**94**		**40**	**45 / 1.8 G**	**5FU**	**-**	**Pros**	**… (9)**
**29**	**92**		**171(39)**	**41.4/1.8 Gy**	**pacli+carbo**	**-**	**CT**	**Van Hagen (12)**
**15.6**	**72**		**60**	**30/2 Gy**	**cis+etop**	**5FU+leu+cis**	**CT**	**… (17)**
**31**	**84.6**		**39**	**35/2.33 Gy**	**5FU+cis**	**-**	**CT**	**Burmeister (10)**
**11.7**	**85**		**34(21)**	**-**	**-**	**doce+cis+5FU**	**CT**	**… (16)**
**16.7**	**100**		**36**	**45 / 1.8 G**	**XELOX**	**-**	**Pros**	**Wong (15)**
**24**	**88**		**61**	**45-54 Gy**	**pacli+carbo**	**pacli+carbo**	**CT**	**Present Study**

**5FU**: 5-flourouracil; **carbo:** carboplatin; **cis:** cisplatin; **CT:** clinical trial; **Cape:** capecitabine; **etop:** etoposide; **Gy:** gray; **leu:** leucovorin; **pacli:** paclitaxel. * From among patients receiving surgery.

As can be seen, studies directly addressing locally advanced proximal gastric and EGJ adenocarcinoma are mainly in the form of pilot or phase-2 clinical trials involving small group of patients. Sample size, in a majority of cases is lower than 50 subjects. They also differ in protocol in terms of applying induction chemotherapy, chemotherapy regimen, and radiotherapy dose (9, 10, 20, 14). Extent of R0 resection and the pathologic complete response observed in this study corresponds with that of earlier studies. Neoadjuvant chemoradiotherapy, regardless of the protocol employed, appears to be associated with high chance of complete surgical resection. Meanwhile, approximately one-fourth of the treated patients exhibited full pathologic response. To expand on the historic role of 5FU and platins in the treatment of gastric and esophageal adenocarcinoma, we must first take a closer look at the earlier studies. 

In the clinical trial carried out by Ajani et al. (21), patients with stomach cancer received neoadjuvant treatment in the form of induction chemotherapy containing cisplatin, FU, and leucovorin before being exposed to chemoradiotherapy with a dose of 45 gray. Of the 34 patients participating in the trial, 28 cases underwent surgery. The extent of R0 resection in that study was reported to be 70% accompanied with a 24% complete pathologic response. In their study conducted in the training universities affiliated to Mashhad University of Medical Sciences, Aledavood et al. applied neoadjuvant chemotherapy with 5FLU regimen to 41 patients with proximal gastric and EGJ adenocarcinoma. Upon completion of chemotherapy, 20 patients were given resection. The results show R0 in almost 80% of patients and a 20% complete pathologic response. In the investigation made by Stahl et al. (17), 60 out of a total of 126 patients studied were randomly chosen to receive neoadjuvant induction chemotherapy (cis, platin, FU, and leucovorin) followed by chemoradiotherapy (cisplatin/etoposide). They reported an R0 resection in 72% and complete pathologic response in 15% of treated patients. 

Studies have also contemplated on the efficacy of taxol-based neoadjuvant treatment regimens in patients with proximal gastric and EGJ adenocarcinomas one of the most prominent all being CROSS studied by Vanhagen et al. (12). Patients with proximal gastric and EGJ adenocarcinoma comprise a significant portion of the population examined (about 70%). CROSS included patients with T3 tumors and less and N0-1 cancer. Hence, they were entirely operable right from the start. In the intervention group, 178 patients received pre-operative weekly chemoradiotherapy with a paclitaxel/carboplatin regimen and a radiotherapy dose of 41.4 gray. The result was an R0 resection in 90% of the cases and a complete pathologic response in almost one-third of the patients. The wide variation in treatment protocols of studies treating proximal gastric and EGJ adenocarcinoma by means of decisive surgery, adjuvant treatment, neoadjuvant chemotherapy or neoadjuvant chemoradiotherapy, makes it difficult to round up on a specific standard treatment procedure. Nonetheless, by due consideration of the aforementioned literature, it appears that neoadjuvant treatment, especially in the form of chemoradiotherapy, is currently the most favored approach. 

Evidently enough, the majority of side effects observed in the course of this study, as a result of administering the treatment protocol applied herein, are of grades 1 and 2, with only about 5% of the patients manifesting grade 3 or side effects Still, the administered dose in concurrent chemotherapy and radiotherapy was adjusted for 40% of the patients by reducing concurrent chemotherapy to 3 courses and less. This change in protocol was put into effect with consideration to reduced tolerance to adverse effects of induction chemotherapy, high age, and a performance status of KPS≤70% among a significant number of patients. In the study conducted by Vanhagen et al. (12), abundance of grade 3 and 4 side effects was reportedly 7%. Not to mention that, in their investigation, 90% of studied patients received all 5 courses of weekly paclitaxel/carboplatin chemotherapy regimen. Also, worth to mention that patient age and performance status in the study was in a far better range compared to that of the present study. By taking into account the similarity observed in the rate of complete pathologic response between neoadjuvant protocols involving and not involving induction chemotherapy, it appears more beneficial to exclude induction chemotherapy in favor of obtaining a higher level of tolerance to concurrent therapy and better realization of radiosensitizing effect. The eminent feature of the current study is its attempt to introduce an alternate and effective treatment regimen for patients with proximal gastric and EGJ adenocarcinoma and applying it to a suitable community of patients as compared to those included in earlier studies. As a single-armed phase-2 clinical trial, the current study is prone to selection bias. At the same time, the single-armed feature diminishes the possibility of direct comparison between different treatment procedures and protocols. 

In addressing future research, it is recommended that studies be conducted in the form of a randomized clinical trial so as to allow direct comparison to be made between the disparate treatment protocols. 

Upon completion of neoadjuvant treatment, 41% of patients underwent surgery that was associated with 20% post-operative adverse effects (including, 1-month mortality and adverse effects requiring repeated surgery) involving 2 deaths after 2 weeks. In the course of surgery, R0 surgical resection was applied to 88% of patients. In the meantime, one-fourth of patients exhibited a full pathologic response to neoadjuvant treatment. This rate of response was not observed in the remaining 75% (55% of whom were relatively responsive and 20% unresponsive). The extent of R0 resection and the scale of pathologic response observed in this research corresponds with that reported in earlier studies. 

Neoadjuvant chemoradiotherapy, regardless of the protocol employed, appears to be associated with complete tumor removal in majority of cases and accompanied with complete pathologic responsiveness of roughly one-fourth of the patients. Moreover, by taking to account the similarity observed in the rate of complete pathologic response in neoadjuvant protocols involving and not involving induction chemotherapy, it appears more beneficial to exclude induction chemotherapy in favor of obtaining a higher level of tolerance to concurrent therapy and better realization of radiosensitizing effect. 

## Funding:

This research was fully funded by the Deputy of Research and Technology, Mashhad University of Medical Sciences (Grant no. 950995).

## Conflict of Interest:

The authors declare that there is no conflict of interest regarding the publication of this paper.
